# Pathological Mechanisms and Therapeutic Targets for Trigeminal Neuropathic Pain

**DOI:** 10.3390/medicines6030091

**Published:** 2019-08-22

**Authors:** Pawan Bista, Wendy L. Imlach

**Affiliations:** Department of Physiology & Monash Biomedicine Discovery Institute, Monash University, Melbourne, VIC 3800, Australia; pawan.bista@monash.edu

**Keywords:** trigeminal neuropathic pain, trigeminal nerve, nociception, pathological pain, nociceptive circuits, orofacial pain, dental pain, migraine, trigeminal spinal caudalis, trigeminal ganglion

## Abstract

Trigeminal neuropathic pain is a chronic pain condition caused by damage or inflammation of the trigeminal nerve or its branches, with both peripheral and central nervous system dysfunction contributing to the disorder. Trigeminal pain conditions present with diagnostic and therapeutic challenges to healthcare providers and often require multiple therapeutic approaches for pain reduction. This review will provide the overview of pathophysiology in peripheral and central nociceptive circuits that are involved in neuropathic pain conditions involving the trigeminal nerve and the current therapeutics that are used to treat these disorders. Recent advances in treatment of trigeminal pain, including novel therapeutics that target ion channels and receptors, gene therapy and monoclonal antibodies that have shown great promise in preclinical studies and clinical trials will also be described.

## 1. Introduction

Trigeminal neuropathic pain, or trigeminal neuralgia, is the pain caused by damage or injury of the somatosensory nervous system originating from the regions of the face as well as within and around the mouth. The presentation of this type of pain may range from dental pain, which is the most common inflammatory pain in the region, to temporomandibular joint disorders (TMDs), trigeminal neuralgia, myofascial pain, headache, neuritis, and idiopathic pain conditions. Neuropathic trigeminal pain can be either episodic or continuous. These conditions are usually a result of injury or disease of one or more nerve roots of the trigeminal ganglion. Possible causes include nerve trauma [1], compression of the trigeminal nerve root as in trigeminal neuralgia [2], demyelinating disorders, such as multiple sclerosis [3], neoplastic infiltration [4], and familial disorders, such as Charcot-Marie-Tooth disease [5], and secondary to herpetic infections [6]. In many cases, the cause of the neuropathic changes are not understood, however it has been suggested that dysfunction of both peripheral or central nervous systems may contribute [2,7,8]. Orofacial pain conditions are difficult to treat in clinical practice and are often confused with dental pain. Understanding the pathophysiology of the disorders is paramount for the management of the pain. 

## 2. Trigeminal Pain Pathways

**Figure 1:**
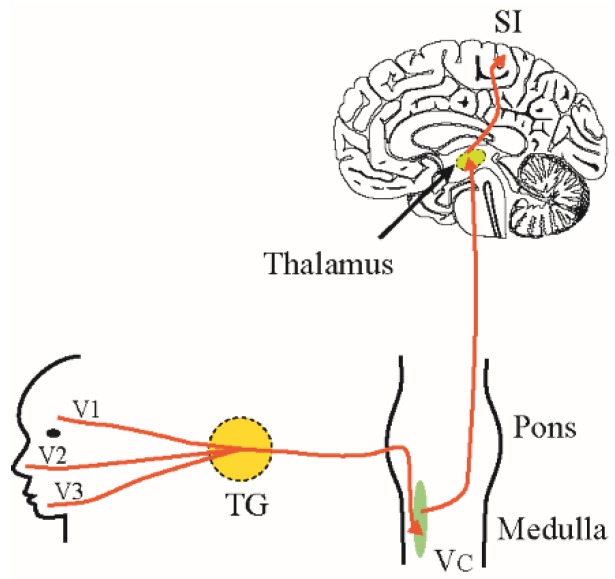
Trigeminal pain pathway. Pain sensation from face and mouth is carried by three peripheral nerve branches (V1, V2 and V3) of trigeminal nerve whose cell bodies sit in trigeminal ganglion (TG) and project centrally to synapse with the second order neurons in the trigeminal spinal nucleus caudalis (V_C_). The second order neurons then ascend to terminate in thalamus. From thalamus, nociceptive information is projected to primary somatosensory cortex (SI) where pain processing occurs. Abbreviations: V1: ophthalmic branch; V2: Maxillary branch; V3; mandibular branch of trigeminal nerve.

Pain sensation in the orofacial region is carried by the trigeminal pathway to the brain. Noxious stimuli such as pinching, prick, hot or cold may activate mechanical and/or thermal nociceptors which are the free nerve endings of the trigeminal sensory afferents. These sensory nerve fibers are myelinated Aδ-fibers and non-myelinated C-fibers whose cell bodies sit in the trigeminal ganglion. Signals are transmitted centrally through these afferent fibers to the trigeminal spinal caudalis (Vc) nucleus of the brain stem where they synapse with second-order neurons that project to the somatosensory and limbic cortices via the thalamus (Figure 1). Throughout this ascending pathway, noxious information is modulated by both local and descending pain modulatory pathways that either inhibit or facilitate the transmitted sensory information [9,10]. Descending inputs to the Vc arise from the primary somatosensory cortex (SI), secondary somatosensory cortex (SII), insula and from the Rostral Ventromedial Medulla (RVM) [8,11,12,13]. Eventually, the interpretation of noxious stimuli is influenced by a number of affective and cognitive factors that modulate pain. Psychological factors like emotion, and state of mind such as attention, understanding, control, expectations and the aversive significance can alter pain perceptions among individuals [14]. Therefore, pain is perceived as a dynamic interaction of cognitive, affective and sensory elements making it a subjective sensation [15,16].

## 3. Therapies Targeting Peripheral Nociceptive Circuit Dysfunction

In some cases, injury or inflammation of orofacial tissues that are innervated by the trigeminal nerve can alter the activity of trigeminal afferent neurons resulting in hyperexcitability, ectopic firing, increased sensitivity to noxious stimuli (hyperalgesia) and innocuous stimuli (allodynia). Peripheral sensitization is characterized by a decreased threshold for activation of transducer channels and an increased firing rate of the sensory neurons leading to hyperalgesia [17]. Sensitization is mediated by a number of inflammatory mediators, neurotrophic factors, neuropeptides at peripheral terminals, as well as differential expression of ion channels, ephatic crosstalk between injured and intact nerve fibers, and increased neurotransmitters release at the central terminals. 

### 3.1. Inflammatory Targets

**Figure 2:**
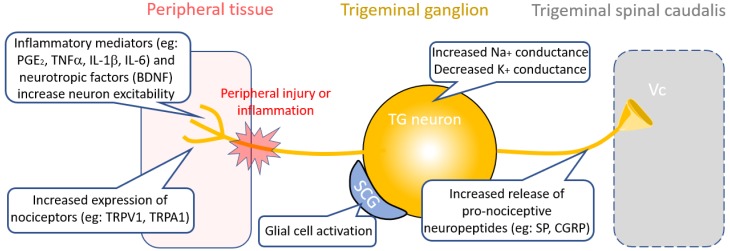
Peripheral mechanisms of trigeminal neuropathic pain. Peripheral nerve injury releases inflammatory mediators including prostaglandin E_2_ (PGE_2_), cytokines and neuropeptides including brain-derived neurotrophic factor (BDNF), which sensitizes peripheral nerve terminals by depolarizing nociceptors. Expression of peripheral receptors including the transient receptor potential vanilloid 1 (TRPV1) and transient receptor potential cation channel, subfamily A, member 1 (TRPA1). Changes in ion channel expression and activity on trigeminal neurons increases excitability as do activated satellite glial cells (SCG), which increase in number. Excitability of the TG neurons leads to increased release of neuropeptides substance P (SP) and calcitonin gene-related peptide (CGRP) to postsynaptic regions in the trigeminal spinal caudalis (brain stem).

Following peripheral nerve-injury, inflammatory mediators including prostaglandin E2 (PGE2), cytokines TNFα, IL-1β, and IL-6 and neurotrophic factors such as brain-derived neurotrophic factor (BDNF) are secreted by activated immune cells including macrophages, neutrophils and mast cells at the site of tissue injury or inflammation [18] (Figure 2). These inflammatory mediators depolarize nociceptive afferents, including the small-diameter trigeminal neurons; increasing neuronal excitability [19]. Therapies designed to prevent the release of inflammatory mediators or inhibit the targets involved can be an effective strategy to treat neuropathic pain. Examples of these include the P2X4 receptor inhibitor Duloxentine, the colony stimulating factor 1 receptor inhibitor PLX5622 and an α4β2 nicotinic acetylcholine receptor (nAChR) agonist TC-2559, which have all been shown to be effective at alleviating neuropathic pain symptoms [20,21,22]. In line with this, a recent study in a rodent model of migraine showed that vagus nerve stimulation effectively inhibited trigeminal nociception through microglial activation via an acetylcholine dependent mechanism [23,24]. Similarly, reduction of pro-inflammatory cytokine signaling by using inhibitors of TNFα, IL-1β, and IL-6 receptors have been shown to attenuate neuronal hypersensitivity after nerve-injury [25,26,27]. Other approaches to target proinflammatory cytokines include intrathecal administration of adeno-associated viral (AAV) vectors that encode the anti-inflammatory cytokine IL-10, or direct injection of recombinant IL-10 into the trigeminal ganglion, which were both found to alleviate neuropathic pain in animal models [28,29].

### 3.2. Neuropeptide Targets

Neuropeptides are a diverse group of molecules that play an important role in pain transmission and modulation. Studies have shown that expression and release of the neuropeptides substance P (SP) and calcitonin gene-related peptide (CGRP) increase in the trigeminal nerve and trigeminal ganglion (TG) in response to peripheral nerve-injury [30] (Figure 2). In recent years, there has been a lot of interest in CGRP as a target for treating trigeminal pain associated with migraine. Therapies targeting CGRP include small molecular CGRP receptor antagonists (the gepants) or monoclonal antibodies (mAbs) to CGRP or its receptor that cause functional blockade of CGRP release. A number of gepants as well as mAbs to CGRP have entered clinical phase trials for drug development [31,32]. The gepant Ubrogepant (MK-1602) is currently under FDA review for acute treatment of migraine and recently two mAbs against CGRP, Galcanezumab and Fremanezumab and an antibody against the CGRP receptor, Erenumab, were approved by the FDA for migraine treatment [33].

### 3.3. Ion Channel Targets

Transmission of sensory information from the periphery to the central nervous system requires coordinated activity of many ion channels and receptors which allow transduction of signals, action potential generation and neurotransmitter release at the synapse. Ion channel dysfunction at all levels of transmission can result in pathological signaling changes, which may contribute to neuropathic pain pathology. Expression and activity of a number of ion channels within pain pathways have been shown to change during the development of trigeminal neuropathic pain. Some of these ion channels contribute to hyperexcitability of TG neurons, including increased activity of Nav1.8 sodium channels due to increased expression [34,35], a reduction in the density of the transient currents (I_A_) and sustained currents (I_K_) of voltage-gated potassium (K^+^) channels [35,36], changes in activation rate and increases in density of currents from hyperpolarization activated cation channels (I_h_) [37] and an increase in transmitter release due to calcium (Ca^2+^) channel activation at central terminals [38,39,40]. Collectively these changes serve to increase the excitability of primary afferents.

#### 3.3.1. TRP Channels

Trigeminal afferents express a number of different types of nociceptors (thermo-, mechano- and chemo- receptors) and expression of many of these receptors has been shown to increase in trigeminal neuropathic pain models [41,42] (Figure 2). Transient receptor potential vanilloid 1 (TRPV1) receptors are polymodal receptors and are activated by various stimuli including noxious heat above 42 °C [43]. Transient receptor potential cation channel subfamily A, member 1 (TRPA1) receptors transmit signals for pain, cold and itch. Expression of both TRPV1 and TRPA1 are increased in Aδ- and C- trigeminal afferents and their thresholds for activation are reduced following nerve-injury [44,45]. Furthermore, trigeminal neuropathic pain injury also leads to over-expression of TRPV1 receptors in large-diameter and uninjured TG neurons leading to the development of mechanical allodynia [45,46]. Similarly, the cold sensing TRP channels TRPA1 and TRPM8 are expressed in trigeminal afferents and TRPA1 also functions as a mechanoreceptor and has been shown to be involved in trigeminal mechanical allodynia [41,42]. Expression of TRPA1 in trigeminal ganglion increases after tooth injury [47], however there has been no evidence to suggest that either TRMP8 and TRPA1 contribute to cold sensitivity in dental pulp [48]. A recent study by Demartini et al. (2018) found that the TRPA1 antagonist ADM_12 treatment alleviated mechanical allodynia in a chronic constriction injury rat model of trigeminal neuropathic pain by inhibiting both TRPA1 and TRPV1 channels [41]. Similarly, in another study, two different TRPV1 channel antagonists namely JNJ-38893777 and JNJ-17203212, were found to reduce trigeminal activation in two migraine rat models, shown as a reduction in c-fos expression and reduced CGRP release [49]. In addition to pharmaceutical approaches, a recent gene therapy study by Guo et al. (2019) using a rat model of orthodontic tooth has shown that reduction of TRPV1 expression in the trigeminal ganglia reduces pain induced by tooth movement in the jaw. In this study, lentivirus containing a TRPV1 shRNA gene was injected into the trigeminal ganglia to reduce TRPV1 protein expression and pain levels were assessed using rat grimace scale (RGS) [50]. RGS is regarded as a proxy to score pain levels in the rats which is assessed by looking the signs of pain from the facial expressions and position of the eyes, ears and mouth of the animal.

#### 3.3.2. Sodium Channels

Overexpression of sodium channels in TG neurons is associated with an increase in neuronal excitability [51]. Voltage gated sodium channels that are involved in action potential generation, such as Nav1.6, have been shown to be linked with gain-of-function mutations causing trigeminal neuropathic pain in patients [52]. The expression of both tetrodotoxin-resistant and -sensitive sodium currents increase in small/ medium diameter TG neurons following trigeminal nerve-injury, leading to hyperexcitation of the TG neurons and induction of mechanical allodynia. Examples of drugs that target sodium channels that are used to treat trigeminal pain are carbamazepine and its derivative oxcarbazepine [53]. These agents act by inhibiting voltage gated sodium channels, thereby reducing the excitability of the neurons [54]. They have also been shown to activate GABA receptors composed of α1, β2, and γ2 subunits in order to inhibit neuronal excitation and ultimately, reduce hyperalgesia and pain [55]. Additionally, in a clinical case series study, intravenous infusion of lidocaine and magnesium relieved pain in patients with trigeminal neuropathic pain by inhibiting sodium channel activity [56].

#### 3.3.3. Potassium Channels and Non-Selective Cation Channels

Damage to trigeminal afferents through chronic constriction in animal models of nerve-injury leads to down-regulation of large-conductance, calcium-activated potassium channels (BK channels), resulting in neuronal hyperexcitability [57]. BK channels regulate the firing pattern of the neuron as they contribute to the afterhyperpolarization, thereby decreasing the firing frequency [58]. Therefore, reduced expression of BK channels in TG after nerve-injury increases excitability and may lead to mechanical allodynia. I_h_ currents also play an important role in the hyperexcitability observed in sensitized peripheral trigeminal afferents. In nerve-injury model of neuropathic pain, I_h_ current density as well as the rate of activation were increased in TG neurons [37]. In line with this finding, blockade of I_h_ attenuated mechanical allodynia and reduced ectopic discharges following nerve-injury [52].

#### 3.3.4. Calcium Channels

When a neuron is activated, the transmitted signals induce an influx of calcium ions at the terminal and triggers release of neurotransmitters. In pathological pain conditions, the excitatory glutamatergic neurons synapses are strengthened increasing the excitability of nociceptive neurons and facilitating the nociceptive transmission to higher brain regions [17,59]. Nociceptive signals can be reduced by inhibiting the calcium dependent neurotransmitter release in the presynaptic compartment through inhibition of N-type voltage-gated Ca^2+^ channels [17,59]. These receptors are the target for gabapentin and pregabalin, which have been shown to be effective at reducing pain in trigeminal neuralgia patients [60,61,62]. Both of these drugs target α2-δ subunit of voltage-gated calcium channels and inhibit release of excitatory neurotransmitters from the presynapse [63,64]. Similarly, botulinum toxin type A also inhibits neurotransmitter release at the presynaptic terminal and has been shown to reduce pain, both inflammatory and neuropathic in origin [65]. A recent clinical trial showed that intradermal injection of botulinum toxin type A was safe and effective at reducing pain in elderly patients with idiopathic trigeminal neuralgia [66].

### 3.4. Glial Targets

Interactions between neurons and glia in the trigeminal ganglion are thought to contribute to the development of neuropathic pain following nerve-injury or inflammation [67]. Satellite glial cells (SGCs) surround TG neurons and modulate activity through the release of neuroinflammatory mediators and neurotransmitters, increasing mechanical hypersensitivity [68,69]. SGCs communicate with each other through gap junctions, which increase in number following nerve-injury [70,71]. Therapeutics designed to inhibit SGCs or their communication with each other and TG neurons may be a feasible approach for the treatment of trigeminal neuropathic pain. In a recent study, minocycline, a glial inhibitor, reduced orofacial nociception by inhibiting SGC mediated release of pro-inflammatory cytokines in the TG [72]. Studies have shown that modulation of specific ion channels and receptors expressed by SGCs, such as the Kir4.1 potassium channel and the purinergic receptor P2Y2 receptor, may be a viable approach for treating trigeminal pain [73,74]. Silencing Kir4.1 channels using siRNA in SGC of the rat TG induced neuropathic pain like behavior in freely moving rats raising the possibility of targeting Kir4.1 to treat neuropathic pain [74]. In a complete Freund’s adjuvant (CFA) model of inflammatory trigeminal pain in the rat temporomandibular joint (TMJ), activated SGCs proliferated and had increased expression of purinergic receptors P2Y1 and P2Y2. The mechanical allodynia that developed in this model could be reduced through administration of the P2Y2 receptor antagonist AR-C118925 due to the inhibition of SGC activation in the TG [73]. Similarly, inhibiting the function of connexin-43 gap junctions using a selective gap junction blocker (Gap27) alleviated mechanical hypersensitivity following inferior alveolar nerve-injury [71]. Gene therapy approaches targeting SGC function has also been described. In a study by Vit et al. (2009), the gene encoding glutamic acid decarboxylase (GAD) was transfected onto SGCs using a serotype 5 adenovirus vector with high tropism for glial cells, resulting in production of the inhibitory neurotransmitter γ-aminobutyric acid (GABA) in the region. The increased inhibitory tone was mediated via neuronal GABAA receptors and was sufficient to produce analgesia resulting from the orofacial inflammatory pain by formalin test [75].

### 3.5. Cross-Excitation from Injured Nerve Fibers

Another peripheral mechanism of trigeminal neuropathic pain that has been described is the cross-excitation from injured nerve fiber to an adjacent intact nerve fiber, which induces allodynia and hyperalgesia in rat models of neuropathic pain [76]. Similarly, biopsy specimens from patients with trigeminal neuralgia show pathological changes, including demyelination and loss of intervening glial cells between groups of axons, which may account for ectopic impulse discharge and cross-excitation among neighboring afferents [77]. A study by Lee et al. (2005) showed that inhibition of I_h_ currents reduced ectopic discharges from Aδ fibers and completely blocked ectopic discharges from Aβ fibers and alleviated allodynia in rat model of nerve-injury induced neuropathic pain [78]. This suggests that HCN channels have potential as a therapeutic target for nerve-injury-induced neuropathic pain.

## 4. Therapies Targeting Central Nociceptive Circuit Dysfunction

Prolonged peripheral nociceptive sensitization may lead to central pathological changes in the trigeminal ascending pain pathways by inducing neuroplastic changes and hyperexcitability of central neurons, and through changes to the pain modulation control by higher brain centers.

### 4.1. Glia in the CNS

Inflammatory mediators in the central nervous system (CNS) play a critical role in sensitization of central nociceptive neurons. Glial cells, namely microglia and astrocytes, proliferate and are activated in response to peripheral nerve injury [79,80] (Figure 2). Activated microglia and astrocytes release chemical mediators that can sensitize nociceptive neurons. Astrocytes are coupled by gap junctions formed by connexin 43 (Cx43) hemichannels. Cx43 hemichannels in astrocytes are implicated in the maintenance of neuropathic pain following peripheral nerve injury. A recent study by Tonkin et al. (2018), showed that a Cx43 mimetic peptide, peptide5, which blocks the hemichannel and reduces microglial and astrocytic activity [81]. This peptide was shown to reduce allodynia in two mouse models of neuropathic pain, including peripheral nerve injury and chemotherapy-induced peripheral neuropathy [81]. Likewise, in another study, application of non-selective gap junction blocker carbenoxolone attenuated facial mechanical hypersensitivity and orofacial neuropathic pain induced by trigeminal nerve injury [82]. 

One of the key functions of astrocytes is to clear excess synaptic glutamate and enzymatically convert it to glutamine with glutamine synthetase. Activated astrocytes release glutamine which is taken up by the primary afferent terminals to replenish the supply of the excitatory neurotransmitter glutamate. More glutamate can then be released at the synaptic cleft allowing increased excitability of the neuron [83]. Chiang et al., (2007) showed that methionine sulfoximine, an inhibitor of the astroglial enzyme glutamine synthetase, strongly attenuated central sensitization induced in functionally identified nociceptive neurons in the Vc. This potent inhibition of central sensitization in an inflammatory mouse pain model was restored by simultaneous intrathecal superfusion of glutamine. This suggest that glutamine synthetase antagonists can induce analgesia and prevent development of neuropathic pain [83]. All these findings point to the important role of neuro-glia communication in the development of orofacial neuropathic pain and suggests this is a viable target to treat such condition.

### 4.2. Inhibitory Neurotransmission in Central Pathways

**Figure 3:**
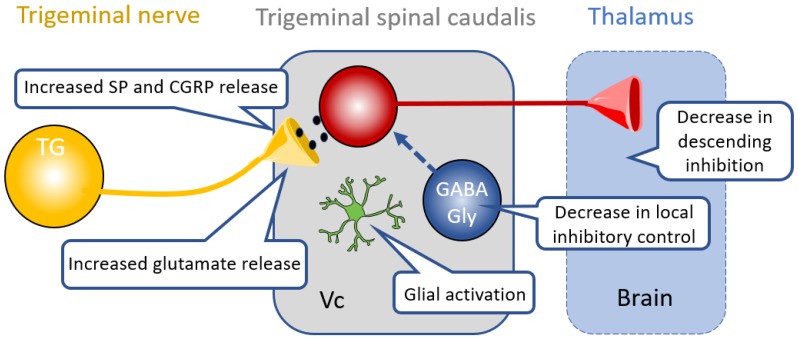
Central mechanisms of trigeminal neuropathic pain. Prolonged and increased nociceptive input from the trigeminal nerve central terminal leads to increased synaptic release of glutamate, Substance P (SP) and calcitonin gene-related peptide (CGRP) in the trigeminal spinal nucleus caudalis (V_C_). This also leads to activation of the glial cells such as astrocytes. Loss of GABA and glycine signaling within local circuits, changes in KCC2 expression and GABA transporters reduce inhibitory control, increasing excitability. Descending inhibitory modulation is also reduced.

Anatomical and functional reorganization of the central pain pathways of the spinal cord have been established in neuropathic pain models [84,85,86]. However, comparatively, few studies have been conducted in understanding neuroplastic changes in CNS of trigeminal neuropathic pain in the rodent. Inhibitory interneurons in the spinal cord and Vc release GABA and/or glycine to decrease the excitability of nociceptive neurons [87]. However, in pathological pain conditions, this inhibition is reduced (disinhibition) resulting in increased excitability in pain pathways (Figure 3). It has been shown in the rodent spinal cord, that disinhibition allows engagement of non-nociceptive Aβ- fibers to the nociceptive circuit such that normally innocuous stimuli are now perceived as painful [85]. Transection of the inferior alveolar nerve (IANX), a branch of V3, is known to produce neuropathic pain symptoms due to hyperexcitation of neurons in the medullary dorsal horn [88]. Increased neuronal excitability and reduced inhibition is also observed in the ascending pain circuits across the thalamus, somatosensory cortex and limbic system which may account for increased orofacial neuropathic pain after trigeminal nerve injury [89]. Following trigeminal nerve injury or inflammation, the trigeminal thalamic, ventral posteromedial nucleus (VPM) neurons showed increased spontaneous activity, larger receptive field size, low activation threshold and therefore, higher excitability [1,90]. Studies are emerging that investigate changes in intracortical as well as local circuit organization leading to cortical hypersensitivity in trigeminal neuropathic pain. A recent study demonstrated facilitation of excitatory propagation but attenuation of inhibitory input to the layer II/III pyramidal neurons in the ventral secondary somatosensory cortex and insular oral region after IANX [89]. These studies highlight the contribution of disinhibition in the development and maintenance of persistent pain. Therapeutics that increase inhibition may be an effective approach to treat trigeminal pain conditions.

Targeting the GABA_A_ receptor or specific subunits of this receptor have shown potential as an analgesic strategy. Two recent studies reported that positive modulation of α6 GABA_A_ receptors subunit potently inhibited trigeminal neuropathic pain. The first report by Vasovic et al. (2019), showed that deuterated pyrazoloquinolinone compound DK-I-56-1 inhibited the development and reduced the established trigeminal neuropathic pain in a rat model of chronic constriction injury of the infraorbital nerve. Moreover, the inhibition was long-lasting and the drug at this therapeutic dose of 10 mg/kg administered intraperitoneally did not affect mouse behavior [91]. In a separate study by Fan et al. (2018), pyrazoloquinolinone compound 6, a positive modulator of α6 GABA_A_ receptors subunit, ameliorated capsaicin-induced vascular effects in an animal model of migraine, indicating that α6 GABA_A_Rs have potential as anti-migraine agents [92]. Even though positive modulation of the GABAA receptor α6 subunit has shown anti-allodynic effect, modulation of GABA_A_ receptor either by its selective antagonist bicuculline or agonist muscimol did not show straightforward effect. In a naive rat, intracisternal administration of bicuculline produced mechanical allodynia while in an inflammatory pain models prepared by subcutaneous injection of interleukin 1 beta (IL-1β), the intracisternal administration of bicuculline produced anti-allodynic effect [93]. It is exactly opposite to what is expected from the action of bicuculline, a GABA_A_ receptor antagonist, which previously has been shown to produce mechanical allodynia when given intrathecally in naïve animal [94]. However, the same drug given via the same route in an inflammatory condition produced opposite (anti-allodynic) paradoxical effect [93]. The anti-allodynic effect observed after intrathecal administration of bicuculline in inflammatory condition also contrasts to what has previously been reported where the study reported that bicuculline administration enhanced formalin induced pain behavior [95]. It is not clear why bicuculline produces opposite effect in naïve versus inflammatory conditions, but this may be due to down-regulation of KCC2, which can lead to inhibitory neurons becoming excitatory [96]. Similarly, the GABA_A_ receptor agonist muscimol depressed mechanically evoked response of Vc nociceptive neurons after intrathecal application in sham rats but not in IANX rats. The negative effect in the IANX rats was due to overall reduction in the number of GABAergic neurons as well as reduced expression of KCC2 in the Vc nucleus which may functionally change the GABAergic signaling from inhibitory to excitatory following inferior alveolar nerve transection [96,97]. 

Another approach to potentiate inhibition is to activate the GABA_B_ receptor. Subcutaneous injection of the GABA_B_ receptor agonist baclofen alleviates the mechanical allodynia-like behavior in a nerve injury rat model of neuropathic pain [98]. It has been shown that peripheral antinociception effect of baclofen is due to the activation of tetraethylammonium-sensitive K^+^ channels [99].

Synaptically released GABA is constantly removed from the synaptic cleft by GABA transporters, namely GAT1 and GAT3, which are present on astrocytes, thus regulating the concentration of inhibitory neurotransmitter at the synaptic junction. In one study it was shown that the expression of GAT-1 and GAT-3 transporters are increased in the spinal trigeminal nucleus in rats with inflammatory pain induced by carrageenan injection. This reduced inhibition of trigeminothalamic neurons in the spinal trigeminal nucleus may contribute to hyperalgesia following carrageenan injections in this rat model [100]. These findings are supported by a study that shows intrathecal injection of a GAT-1 inhibitor attenuates paclitaxel-induced neuropathic pain. Paclitaxel is a chemotherapeutic agent which often results in the development of neuropathic pain when administered to both patients as in animal models of chemotherapy-induced pain [101]. Another approach to increase inhibition in pain pathways is by activating glycinergic inhibitory neurotransmission in the spinal trigeminal nucleus. Glycinergic neurons and their receptors are densely distributed in medullary dorsal horn and play an important role to maintain physiological levels of pain sensitivity together with GABAergic inhibition [102,103]. Dysfunction of glycinergic inhibition has been shown to produce mechanical allodynia, a hallmark of orofacial neuropathic pain [104]. Under physiological condition, glycinergic inhibition gates tactile input to nociceptive neurons in MDH through a local circuit involving neurons expressing the gamma isoform of protein kinase C (PKCγ). However, under pathological neuropathic pain condition, the glycinergic feedforward inhibition to PKCγ-positive neurons was shown to be reduced (disinhibition) and innocuous mechanoreceptive pathways were engaged that activated nociceptive circuits [104]. Pharmacological therapies directed to potentiate glycinergic inhibition might provide a new tool for alleviating allodynia and neuropathic pain [105].

### 4.3. Descending Modulation

Neuroplastic changes occur not only in the sensory ascending pain pathway but also in the descending modulatory pathways [11,106,107,108] (Figure 3). A wide variety of neurotransmitters including GABA, serotonin, adrenaline, dopamine, acetylcholine and nitric oxide are involved in modulating pain signals. The descending pathways from primary somatosensory cortex and medulla project directly to target neurons in the Vc to suppress responses to noxious stimuli and produce behavioral hypoalgesia [109,110]. Efferents from the somatosensory cortex have been shown to produce analgesia by feedforward inhibition of projection neurons in the Vc through GABA_A_ receptor-mediated signaling [109]. Modulation of nociceptive signals from the RVM produces bidirectional pain modulation either by inhibiting or enhancing nociceptive inputs [111,112]. Drugs that target this system include the tricyclic antidepressants (TCAs), including amitriptyline, nortriptyline and desipramine, which inhibit norepinephrine reuptake [113,114]. In addition to pharmacotherapy, neurostimulation in the form of deep brain stimulation, or transcranial brain stimulation of the descending pain pathway have been effectively used to inhibit primary headache as well as trigeminal pain in patients (Table 1). A recent study provided direct evidence that electrical stimulation of the somatosensory cortex can activate inhibitory neurons in the VC, thereby producing strong feedforward inhibition of projection neurons and excitatory neurons in the nucleus leading to behavioral hypoalgesia [109,115,116].

## 5. Conclusions and Future Perspectives

Trigeminal neuropathic pain is a debilitating chronic pain condition occurring after peripheral trigeminal nerve injury or inflammation, with symptoms that include hyperalgesia and mechanical allodynia. There are many potential mechanisms underlying this condition—ranging from cross-excitation of intact nerve by injured nerve, neuro-glial interactions, wiring changes in the CNS, loss of inhibitory control, change in ion channel function, and sensitization by inflammatory mediators. Thus, these symptoms arise because of the defects in pain processing pathway rather than noxious stimuli and deficits in the peripheral and central nervous system function contribute to the development and maintenance of these conditions. Much of our understanding of neuropathic pain processing comes from experiments in spinal cord dorsal horn, but comparatively fewer studies have been conducted in trigeminal pathway. There is still a huge knowledge gap in the central neuroplastic changes that occur after trigeminal nerve injury, which remains a major challenge to tackle in the coming years. Defects in several types of ion channels, cell types including both neurons and glia as well as neuronal circuits have been shown to be involved in processing the nociceptive information and are therefore potential therapeutic targets for the treatment of trigeminal neuropathic/inflammatory pain (Table 1).

## Figures and Tables

**Table 1:** Clinical and pre-clinical therapeutics for the treatment of trigeminal neuropathic pain.

Clinical Condition or Animal Model	Therapeutic Target	Therapeutic Agent	Effect on Symptoms, or Behavioral Outcome	Reference
**Peripheral Nociceptive Pathways**				
Infraorbital nerve constriction model, rat	IL-10 gene expression in glial cells	Viral vector (AAV) encoding IL-10	Suppresses mechanical allodynia and thermal hyperalgesia	Iwasa et al., 2019; Milligan et al., 2005 [28,29]
Phase III clinical trial for migraine	CGRP receptor (antagonist)	Erenumab	Anti-migraine	Goadsby et al., 2017; Traynor K, 2018 [31,33]
Phase III clinical trial for migraine	CGRP receptor (antagonist)	Gepants (Ubrogepant, Rimegepant)	Anti-migraine	Holland PR, 2018 [32]
α-CGRP intra-TG injection model, rat	Glial cell (inhibitor)	Minocycline	Reduces thermal hyperalgesia	Afroz et al., 2019 [72]
TMJ inflammation induced by CFA injection, rat	P2Y2 receptor (antagonist)	AR-C118925	Reduces mechanical allodynia	Magni et al., 2015 [73]
Inferior alveolar nerve transection (IANX) model, rat	Cx43 gap junctions	Gap27 (Cx43 blocking peptide)	Attenuates mechanical hypersensitivity	Kaji et al., 2016 [71]
Orofacial formalin model, rat	GAD65 gene expression (in SGC)	Viral vector (AAV) encoding GAD	Blocks pain behaviour (orofacial rub)	Vit et al., 2009 [75]
Clinical study of idiopathic trigeminal neuralgia in elderly (≥ 80 years) or adult > 60 years old patient groups	Acetylcholine release (inhibitor)	Botulinum Toxin Type A	Relief of trigeminal pain symptoms	Jing et al., 2018 [66]
**Central Nociceptive Pathways**				
Clinical case studies in 9 patients with intractable trigeminal neuralgia	NaV channels (antagonist)	Intravenous magnesium and lidocaine	Relief of trigeminal pain symptoms	Arai et al., 2013 [56]
Partial infraorbital nerve transection model, rat	Gap junction (blocker)	Carbenoxolone	Reduces facial mechanical hypersensitivity and central sensitization	Wang et al., 2014 [82]
Allyl isothiocyanate tooth pulp inflammation model, rat	Astroglial enzyme glutamine synthetase (inhibitor)	Methionine sulfoximine	Reduces central sensitization	Chiang et al., 2007 [83]
Chronic constriction injury of the infraorbital nerve, rat	α6 GABA_A_R	DK-I-56-1	Reduces mechanical hypersensitivity	Vasovic et al., 2019 [91]
Subcutaneous injection of IL-1β, rat	GABA_A_R (antagonist)	Bicuculline	Allodynia in naïve rat and anti-allodynic effect in IL-1β injected rat	Kim et al., 2017 [93]
Inferior alveolar nerve transection (IANX) model, rat	GABA_A_R (agonist)	Muscimol	Decreases mechanical evoked response	Okada-ogawa et al., 2015 [97]
Chronic inferior alveolar nerve constriction, rat	GABA_B_R (agonist)	Baclofen	Reduces mechanical allodynia like behaviour	Idanpaan-Heikkila et al., 1999; Reis et al., 2006 [98,99]
Primary headache, Intractable facial pain, in clinical use	Vagus nerve, cortex (transcranial stimulation). Occipital nerve, ventral tegmental area (invasive stimulation)	Neurostimulat-ion through transcranial magnetic stimulation or invasive brain stimulation	Reduction in headache symptoms, reduced facial pain	Miller et al., 2016 [115] Osenbach, 2006 [116]
**Ion Channel Targets**				
Chronic constriction injury of the infraorbital nerve, rat	TRPA1 (antagonist)	ADM_12	Reduction of mechanical allodynia	Demartini et al., 2018 [41]
Orthodontic pain model, rat	TRPV1 receptors	Lentivirus delivery of shRNA for TRPV1	Reduces pain from tooth movement	Guo et al., 2019 [50]
Phase III clinical trial for trigeminal neuralgia, human	Voltage dependent calcium channel (inhibitor)	Gabapentin	Reduces neuropathic pain and trigeminal neuralgia pain	Yuan et al., 2016; Serpell et al., 2002 [60,62]
Phase III clinical trial for trigeminal neuralgia, human	Voltage dependent calcium channels (inhibitor)	Pregabalin	Reduces trigeminal neuralgia related pain	Obermann et al., 2008 [61]
